# Understanding the Dynamics of the Transient and Permanent Opening Events of the Mitochondrial Permeability Transition Pore with a Novel Stochastic Model

**DOI:** 10.3390/membranes12050494

**Published:** 2022-04-30

**Authors:** Keertana Yalamanchili, Nasrin Afzal, Liron Boyman, Carmen A. Mannella, W. Jonathan Lederer, M. Saleet Jafri

**Affiliations:** 1 School of Systems Biology, George Mason University, Fairfax, VA 22030, USA; keertanay6@gmail.com (K.Y.); nasrin.afzal@gmail.com (N.A.); 2 Thomas Jefferson High School for Science and Technology, Alexandria, VA 22312, USA; 3 Center for Biomedical Engineering and Technology, University of Maryland School of Medicine, Baltimore, MD 21201, USA; lboyman@som.umaryland.edu (L.B.); cmannella@som.umaryland.edu (C.A.M.); jlederer@som.umaryland.edu (W.J.L.)

**Keywords:** reactive oxygen species (ROS), mitochondria, computational model, heart, mPTP, Monte Carlo method, depolarization, TMRM

## Abstract

The mitochondrial permeability transition pore (mPTP) is a non-selective pore in the inner mitochondrial membrane (IMM) which causes depolarization when it opens under conditions of oxidative stress and high concentrations of Ca^2+^. In this study, a stochastic computational model was developed to better understand the dynamics of mPTP opening and closing associated with elevated reactive oxygen species (ROS) in cardiomyocytes. The data modeled are from “photon stress” experiments in which the fluorescent dye TMRM (tetramethylrhodamine methyl ester) is both the source of ROS (induced by laser light) and sensor of the electrical potential difference across the IMM. Monte Carlo methods were applied to describe opening and closing of the pore along with the Hill Equation to account for the effect of ROS levels on the transition probabilities. The amplitude distribution of transient mPTP opening events, the number of transient mPTP opening events per minute in a cell, the time it takes for recovery after transient depolarizations in the mitochondria, and the change in TMRM fluorescence during the transition from transient to permanent mPTP opening events were analyzed. The model suggests that mPTP transient open times have an exponential distribution that are reflected in TMRM fluorescence. A second multiple pore model in which individual channels have no permanent open state suggests that 5–10 mPTP per mitochondria would be needed for sustained mitochondrial depolarization at elevated ROS with at least 1 mPTP in the transient open state.

## 1. Introduction

The mitochondrial permeability transition pore (mPTP) is a non-specific, transmembrane structure in the inner mitochondrial membrane (IMM) that normally resides in the closed state. Several external factors can cause the pore to switch from the closed state to the open state. The opening can be transient or permanent. Previous studies in isolated mitochondria have suggested that conditions of oxidative stress, depletion of adenine nucleotides, and/or high concentrations of matrix Ca^2+^ ([Ca^2+^]_m_) underlie the mPTP opening events [1]. Oxidative stress occurs when the rate of production of reactive oxygen species (ROS) is greater than the rate at which ROS is detoxified, resulting in elevated ROS levels in the mitochondria. This leads to mPTP opening events [2]. 

The mPTP opening events are considered to have major implications for several biological processes and conditions. For example, permanent mPTP opening has been associated with cell death [3,4]. There are two types of mPTP opening events: transient and permanent. In transient opening events, the pore switches from the closed state to the transient open state and then back to the closed state, while permanent opening events switch the pore to the permanent open state. On a cellular level, permanent mPTP opening events depolarize the IMM, inhibit ATP synthesis and possibly induce cell death. Previous research has shown that when mPTP is in the permanent open state, mitochondria swell, eventually causing necrotic cell death [5]. Conversely, closed mPTPs maintain the membrane potential and integrity of the IMM, preserving homeostatic conditions in the mitochondrial matrix [6]. Once the mPTP opens permanently, the mitochondrion is no longer intact and the IMM is irreversibly depolarized. Since the membrane potential of the IMM drives ATP synthesis, the depolarization caused by permanent mPTP opening stops any and all ATP synthesis. 

Although several implications of mPTP have been reported, the behavior of mPTP and the transition between transient and permanent opening events still pose unanswered questions. In this study, a computational model was developed to describe the opening and closing events of the mitochondrial permeability transition pore using recent experimental data from isolated heart muscle cells (cardiac ventricular myocytes) in Boyman et al. (2019) [7]. These data were chosen to constrain the model since it is one of the few studies of mPTP in functioning mitochondria in living cells and the experimental manipulations were minimal so as not to perturb normal function. The model includes TMRM (tetramethylrhodamine methyl ester), a potentiometric fluorescent dye, as an indicator of mPTP opening events, according to the experimental protocol of Boyman et al. (2019). Along with better understanding the correlation between single and multiple mPTP opening events, another major purpose of this model was to augment the interpretation of the mPTP dynamics beyond what was experimentally measurable. Particularly, the model would verify how the sampling rate in the experimental study impacted the experimental data, including whether mPTP transient opening events truly have different amplitudes. The application of Monte Carlo methods simulates the data observed in the experimental study. The usage of the Hill Equation characterizes any cooperativity in the impact of ROS on the opening and closing events of mPTP. Furthermore, Euler’s method was used to linearly approximate the reaction rates of the transitions from the closed state to the transient open state, the transient open state to the closed state, and the transient open state to the permanent open state. In addition, the model incorporated the addition of two different types of inhibitors of mPTP opening, cyclosporine-A (CsA) and n-acetyl cysteine (NAC). 

## 2. Materials and Methods

### 2.1. mPTP Model

**Figure 1:**
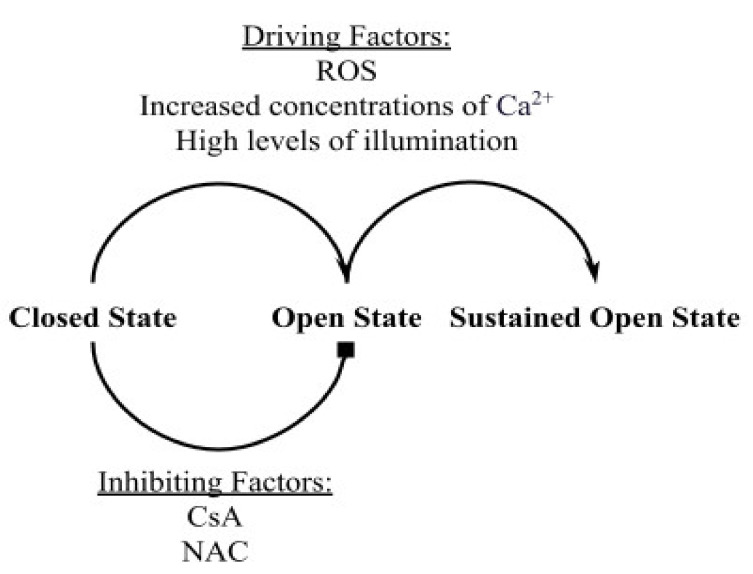
A schematic drawing showing the different elements driving and inhibiting the opening of the mitochondrial permeability transition pore (mPTP). Cyclosporin A (CsA) inhibits pore opening by reducing its sensitivity to driving factors. N-acetyl cysteine (NAC) is a reactive oxygen species (ROS) scavenger that reduces the number of opening events by lowering cellular ROS levels.

Data from the experimental study of Boyman et al. [7] constrained the computational model. Figure 1 represents the different factors that drive mPTPs to transition from one state to another. Reactive oxygen species (ROS) from various sources and increased concentrations of Ca^2+^ in the mitochondrial matrix ([Ca^2+^]_m_) can induce mPTP activation, causing it to switch from the closed state to the transient open state or from the transient open state to the permanent open state. Along with ROS and increased concentrations of Ca^2+^, high levels of laser illumination can increase mPTP activation because fluorescent dyes such as TMRM, used to measure the potential across the inner mitochondrial membrane (IMM), produce ROS by photooxidation [7]. In order to attenuate the effect of the light intensity on mPTP activation, the experimental study used low levels of illumination, which lowered the amount of ROS produced by “photon stress” [7]. The model included two inhibitors used in the experimental study, both of which lower the probability of mPTP transitioning from the closed state to the transient open state: cyclosporin A (CsA), which reduces sensitivity of mPTP to the driving factors and N-acetyl cysteine (NAC), a ROS scavenger that lowers cellular ROS levels. 

Based upon the experimental observation the model assumes that mPTP has three states: the closed state, the transient open state, and the permanent open state. The mPTP state diagram is as follows:

(1) $$\mathrm{Closed}\;\mathrm{State}\underset{Q}{\overset{P}{⇌}}\;\mathrm{Transient}\;\mathrm{Open}\;\mathrm{State}\;\overset{Z}{\to }\;\mathrm{Permanent}\;\mathrm{Open}\;\mathrm{State}$$

where P is the transition probability from the closed state to open state, Q is the transition probability from the transient open state to the closed state, and Z is the transition probability to the permanent open state from the transient open state. The transition probabilities are based on the transition rates described by the Hill equation to establish the rate-dependence on ROS and calcium (Equation (2)). In the equation, V_max_ represents the maximum reaction velocity, [S] is the substrate concentration, n is the Hill coefficient, and K is the Michaelis constant.

(2) $$V=\frac{V_{\max}{\left[S\right]}^{n}}{K^{n}+{\left[S\right]}^{n}}$$

The mPTP has been shown to increase its open probability in response to increased ROS [8]. Moreover, antioxidant compounds or ROS scavengers reduce mPTP opening [7]. The model reflects this by the Hill equation (in µM) with parameters (n—cooperativity and K—binding constant) adjusted to reproduce the Boyman et al. data.

(3) $$\mathrm{ROS}\;\mathrm{dependent}\;\mathrm{opening}\;\mathrm{rate}=\frac{{\left[\mathrm{ROS}\right]}^{4}}{{\left[0.1\right]}^{4}+{\left[\mathrm{ROS}\right]}^{4}}$$

The values of n = 4 and K = 0.1 µM were fitted to simulate the experimentally measured transient opening. The V_max_ is omitted here, but it is included in the final transition probability in Equation (6). Transitions to the permanent open state were also assumed to be ROS-dependent and were also modeled with a Hill equation, with the values for the cooperativity (n = 4), Michaelis constant (K = 0.5 µM) and maximal opening rate (V_max_ = 0.001 s^−1^) fitted to match the experimentally measured permanent opening events.

(4) $$\mathrm{ROS}\;\mathrm{dependent}\;\mathrm{permanent}\;\mathrm{opening}\;\mathrm{rate}=\frac{0.001{\left[\mathrm{ROS}\right]}^{4}}{{\left[0.5\right]}^{4}+{\left[\mathrm{ROS}\right]}^{4}}$$

**Figure 2:**
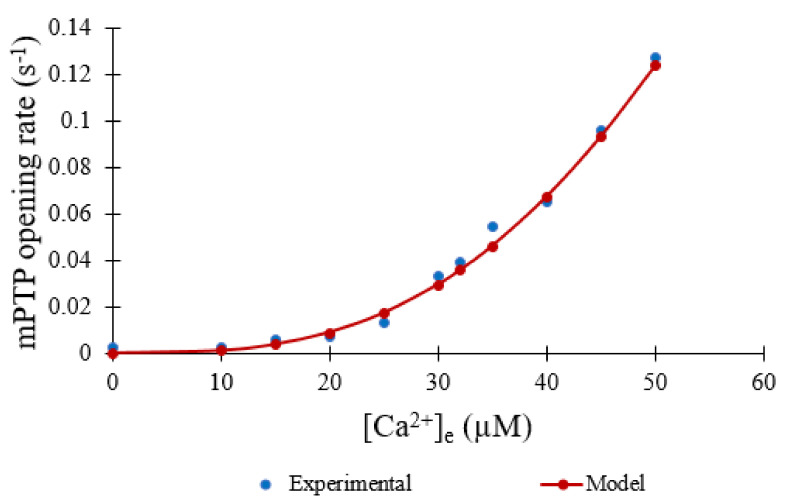
Calcium dependence of mPTP opening based on the assumption that the normalized rate of mPTP opening is linearly proportional to the rate of experimentally measured volume change (blue) [9]. Model fit to data (red).

Experimental studies conclude that the mPTP has an increased open probability in response to elevated extramitochondrial [Ca^2+^]_e_. However, experimental quantitative data measuring this is limited. In experiments, Clarke et al. used changes in mitochondrial volume (measured by light scattering) to assess the extent of mPTP opening (both transient and sustained) [9]. Within the context of the experiments, we assume that the rate of shrinkage of preswollen mitochondria is proportional to the fraction of mitochondria with at least 1 open pore. In the current study, the Ca^2+^ is fixed so this representation will suffice until better data becomes available. This and other data used for these modeling studies were digitized using the Engauge Digitizer (https://markummitchell.github.io/engauge-digitizer/—last accessed 20 December 2021) and normalized. The binding constant and cooperativity were fitted with the following Hill equation (in µM) using the online curve-fitting tool at https://mycurvefit.com/ (last accessed on 20 December 2021) (Figure 2):

(5) $$\;\mathrm{calcium}\;\mathrm{dependent}\;\mathrm{opening}\;\mathrm{rate}=\frac{{\left[{\mathrm{Ca}}^{2+}\right]}^{3}}{{\left[96\;µM\right]}^{3}+{\left[{\mathrm{Ca}}^{2+}\right]}^{3}}$$

The V_max_ is omitted in the normalized equation but will be accounted for in Equation (6).

The opening transition probability P (Equation (6)) is the product of the Ca^2+^-dependent opening rate and the ROS-dependent opening rate multiplied by the integration time step dt. The maximal rate $8.25\times 10^{-7}$ fits the opening rate to the experimental data and captures both the rates due to ROS and Ca^2+^. The value of [Ca^2+^]_e_ was assumed to be at the physiological concentration of 0.1 µM. Multiplying the closing rate of 0.1 s^−1^, which matches the experimental average open time of 10 s, by dt yields the closing transition probability Q (Equation (7)). The transition probability to the permanent open state Z was also ROS dependent to match the experimental data on permanent openings (Equation (8)).

(6) $$P=\frac{8.25\times 10^{-7}{\left[{\mathrm{Ca}}^{2+}\right]}_{e}^{3}}{{\left[96\right]}^{3}+{\left[{\mathrm{Ca}}^{2+}\right]}_{e}^{3}}\frac{{\left[\mathrm{ROS}\right]}^{4}}{0.1^{4}+{\left[\mathrm{ROS}\right]}^{4}}\mathrm{dt}$$

(7) Q=0.1dt

(8) $$Z=\frac{0.001{\left[\mathrm{ROS}\right]}^{4}}{0.5^{4}+{\left[\mathrm{ROS}\right]}^{4}}\mathrm{dt}$$

### 2.2. Mitochondrial Membrane Potential (ψ)

In the model, the mitochondrial membrane potential (ψ) is set to −180 mV at rest. With mPTP opening, the membrane potential depolarizes. This is based on the modeling studies by Nguyen et al., where the opening and closing of an ion channel, with a large ion conductance similar to mPTP, results in step-like changes in the mitochondrial membrane potential [10,11].

### 2.3. TMRM

TMRM is a positively charged fluorescent dye that crosses the polarized mitochondrial membrane in a concentration dependent manner [12]:

(9) $${\left[\mathrm{TMRM}\right]}_{\mathrm{out}}\underset{k_{\mathrm{out}}}{\overset{k_{\mathrm{in}}}{⇌}}\;{\left[\mathrm{TMRM}\right]}_{\mathrm{in}}$$

For simplicity, the assumption of mass action kinetics yields the following dynamic equation to describe the change in concentration of TMRM inside mitochondria ([TMRM]_in_):

(10) $$\frac{d{\left[\mathrm{TMRM}\right]}_{\mathrm{in}}}{\mathrm{dt}}=\left(\left({\left[\mathrm{TMRM}\right]}_{\mathrm{Total}}-{\left[\mathrm{TMRM}\right]}_{\mathrm{in}}\right)\times k_{\mathrm{in}}-{\left[\mathrm{TMRM}\right]}_{\mathrm{in}}\times k_{\mathrm{out}}\right)$$

where [TMRM]_Total_ is the total TMRM concentration, k_in_ is the rate constant for TMRM entry (influx) into the mitochondria, and k_out_ is the rate constant for TMRM efflux. The influx rate constant is

(11) $$k_{in}=0.0002\times \mathrm{abs}\left(\psi \right)$$

where abs(ψ) is the absolute value of the mitochondrial membrane potential ψ. The remaining parameters are given in Table 1.

### 2.4. ROS Production

Photoexcitation of TMRM has been shown to produce singlet oxygen which can quickly be converted to any of the common ROS species [13,14,15,16,17]. A minimal model was developed to describe ROS dynamics. The ROS concentration ([ROS]) is assumed to be 0.01 µM under resting conditions. This study assumes that once the experiment starts, ROS will be produced by the interaction of the applied imaging laser and TMRM in a continuous manner with rate k_ros._ The removal of ROS by a buffering system is assumed to be fast. It is modeled using the rapid buffering approximation

(12) $$\beta_{\mathrm{ROS}}=\frac{1}{\left(1+\frac{{\left[\mathrm{BUF}\right]}_{\mathrm{Total}}K_{\mathrm{BUF}}}{{\left(K_{\mathrm{BUF}}+\left[\mathrm{ROS}\right]\right)}^{2}}\right)}$$

where [BUF]_Total_ is the total ‘concentration’ or capacity of the buffering system and K_BUF_ is the equilibrium constant for the interaction of ROS with the ROS buffering system [18]. The dynamic equation for [ROS] is described by

(13) $$\frac{d\left[\mathrm{ROS}\right]}{\mathrm{dt}}=\beta_{\mathrm{ROS}}k_{\mathrm{ROS}}$$

### 2.5. Numerical Methods

The differential equation describing TMRM dynamics was solved using explicit Euler methods. The model was written in FORTRAN and the figures generated were produced using Excel. The Monte Carlo simulation methods are described below. Model code is provided in Appendix A.

### 2.6. Markov Chain Monte Carlo Method

**Figure 3:**
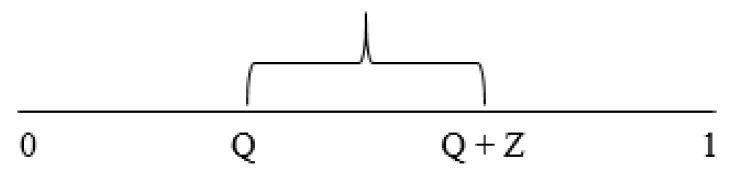
Schematic diagram showing the tiling of the number line for the Markov Chain Monte Carlo Simulation.

The Markov chain Monte Carlo method is used to reproduce the stochastic transition between states in the mPTP model. The opening and closing of mPTP is assumed to obey the Markov property—that the reachable states and the probabilities of reaching those states are only dependent on the current state and no other previous states. In this study, this method was used as a means of representing the randomly determined transitions between the closed state, the transient open state, and the permanent open state of the mPTP. A uniform pseudorandom number on the interval [0, 1) was generated on the computer and compared to the transition probabilities. For example, when the pore is in the transient open state, there is a transition probability Q that it will transition into the closed state (Equation (7) above) and a transition probability Z (the difference between Q + Z and Q, as shown by the bracket in Figure 3) that it will transition into the permanent open state (Equation (8) above). These are used to tile the number line [0, 1]. The probability of staying in the transient open state is 1 − (Q + Z). This means that the rate at which mPTP transitions from the transient open state to the permanent open state is positive and less than 1, even while accounting for the rate at which mPTP transitions back from the transient open state to the closed state. The same approach is used for when the pore is in the closed state, with the probability that it will transition to the transient open state, P, given by Equation (6) (above). The probability that the pore will transition out of the permanent open state is considered zero. 

## 3. Results

**Figure 4:**
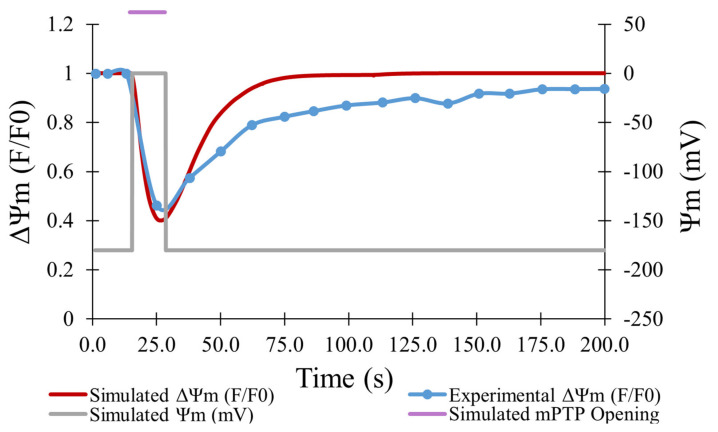
Average change in TMRM fluorescence (F/F0) over time for transient opening events. Experimental TMRM fluorescence for 478 transient opening events (blue) [7]. Model fit to data with 500 transient opening events (red). Mitochondrial membrane potential during an average transient opening event (gray—right axis) was assumed to exactly follow the timing and duration of an average transient opening event.

In the experiments of Boyman et al., laser irradiation of myocytes containing TMRM resulted in efflux of the dye from mitochondria, consistent with depolarization caused by ROS-induced opening of the mPTP [7]. As shown in Figure 4, the experimental data (blue line) showed a maximum decrease in TMRM fluorescence of 54% on average for 478 transient opening events over 180 s, which was matched well by the model for 500 simulated openings (red line). Data was fit using an average transient opening event that started at the 19-s mark and lasted for a duration of 14 s. During this event, depolarization of the mitochondrial membrane potential was presumed to drop from −180 mV to 0 mV. 

**Figure 5:**
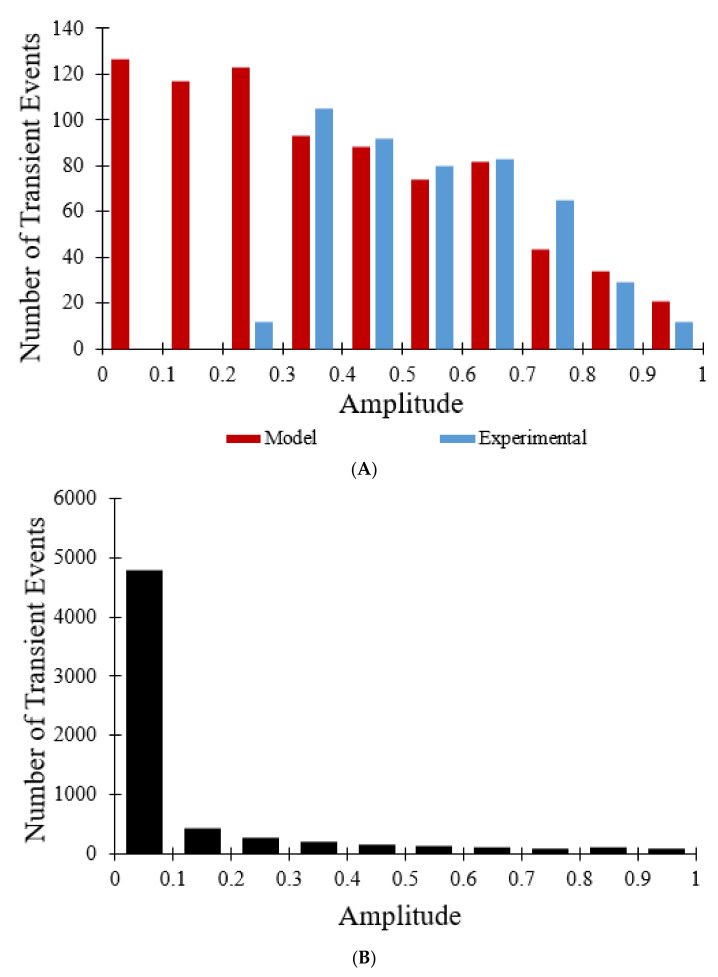
Amplitude distribution for transient opening events. (**A**) Experimental distribution of amplitudes of 478 transient opening events (blue) [7]. Model distribution of amplitudes of 800 transient opening events with a 10 s sampling rate fit to data (red). (**B**) Model distribution of amplitudes of 6334 transient opening events with a sampling rate less than 0.1 s.

Figure 5A is a histogram of the amplitude distribution of transient opening events. The amplitudes ranged from 0 to 1, with decreasing numbers of events for increasing amplitude. The model distribution (for 800 events) closely aligns with the experimental data for 478 events with amplitudes greater than 0.3 but contains many more events at lower amplitudes [7]. When the 10 s sampling rate used experimentally was changed to less than 0.1 s the amplitude histogram for all transient opening events changed considerably. As shown in Figure 5B, 6334 transient opening events were observed over the same time interval (60 s), as opposed to 800 transient opening events, indicating that the larger sampling interval reduces detection of transient events with durations less than the sampling rate. The magnitude of the TMRM fluorescence drop is positively correlated with the pore open time. In addition, although Figure 5A indicates that most of the transient opening events have amplitudes between 0.3 and 0.8, Figure 5B reveals that out of 6334 total transient opening events, 4784 of the events have amplitudes between 0 and 0.1, showing that the sampling rate not only has an impact on the number of transient opening events observed but also on the amplitude distribution of the transient opening events. 

**Figure 6:**
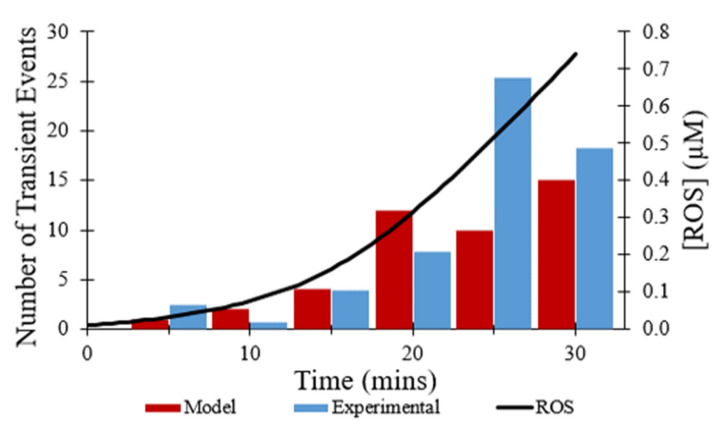
Frequency of transient openings per cell. Number of experimental transient depolarizations per cell per minute at times indicated (blue) [7]. Number of modeled transient depolarizations per cell per minute fit to data (red). Change in ROS concentration during time course of modeling (black). The event frequency at each time point was based on 50 total events.

Figure 6 shows the number of transient opening events per cell per minute over 30 min, as shown in red, which was meant to model the experimental data shown in blue [7]. In both the modeled and experimental datasets, frequency of the transient opening events increases as [ROS] rises (Figure 6—black line). The number of transient opening events decreases at later times as more pores enter the permanent open state (Figure 10C). 

**Figure 7:**
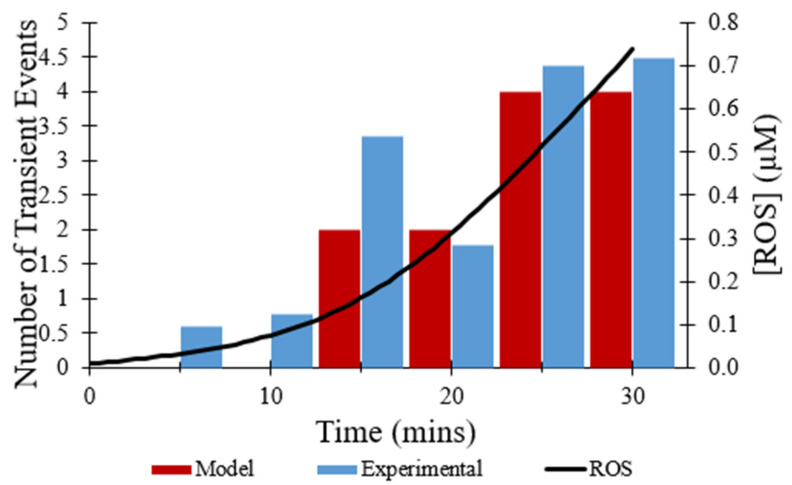
Frequency of transient openings per cell with the addition of CsA. Number of experimental events per cell per minute at times indicated (blue) [7]. Number of modeled events per minute per cell fit to data (red) with the addition of 10 mM CsA. The change in ROS concentration during time course of modeling (black). The event frequency at each time point was based on 50 total events.

The effect of the direct inhibitor of mPTP, CsA, on the observed frequency of transient opening events [7] was simulated by multiplying the rate constant for transitions from the closed to transient open state by 0.05. As depicted in Figure 7, the total number of transient opening events over 30 min decreased from 44 events to 15 events with the presence of CsA, a 66% decrease. While the addition of CsA does reduce the total number of transient opening events over time, the frequency of transient openings increases with buildup of ROS, as expected. 

**Figure 8:**
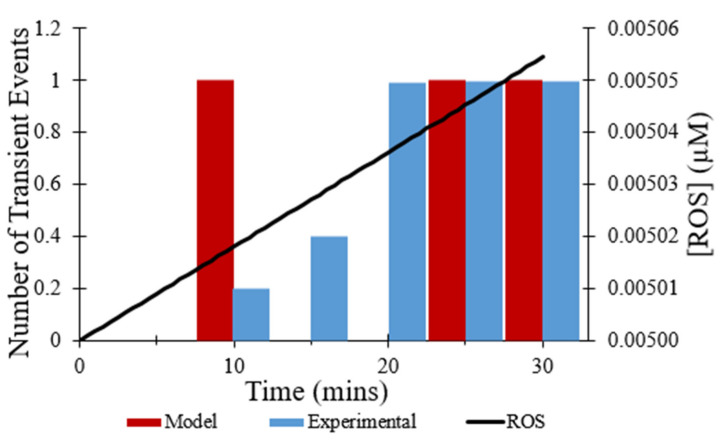
Frequency of transient openings per cell with the addition of NAC. Number of experimental events per cell per minute at times indicated (blue) [7]. Number of modeled events per minute per cell fit to data (red) with the addition of 10 mM NAC. ROS concentration during time course of modeling is essentially flat (black). The event frequency at each time point was based on 50 total events.

In addition to CsA, the effect of the ROS scavenger NAC on the observed frequency of transient opening events [7] also was replicated in modeling. The addition of NAC was mimicked by changing the rate at which ROS levels increased, since NAC scavenges ROS. As depicted in Figure 8, the total number of transient opening events over 30 min decreased from 44 events without the addition of NAC to 3 events with the presence of NAC, an 80% decrease. ROS levels were essentially flat over time resulting in the low frequency of transient opening events throughout the experiment. 

**Figure 9:**
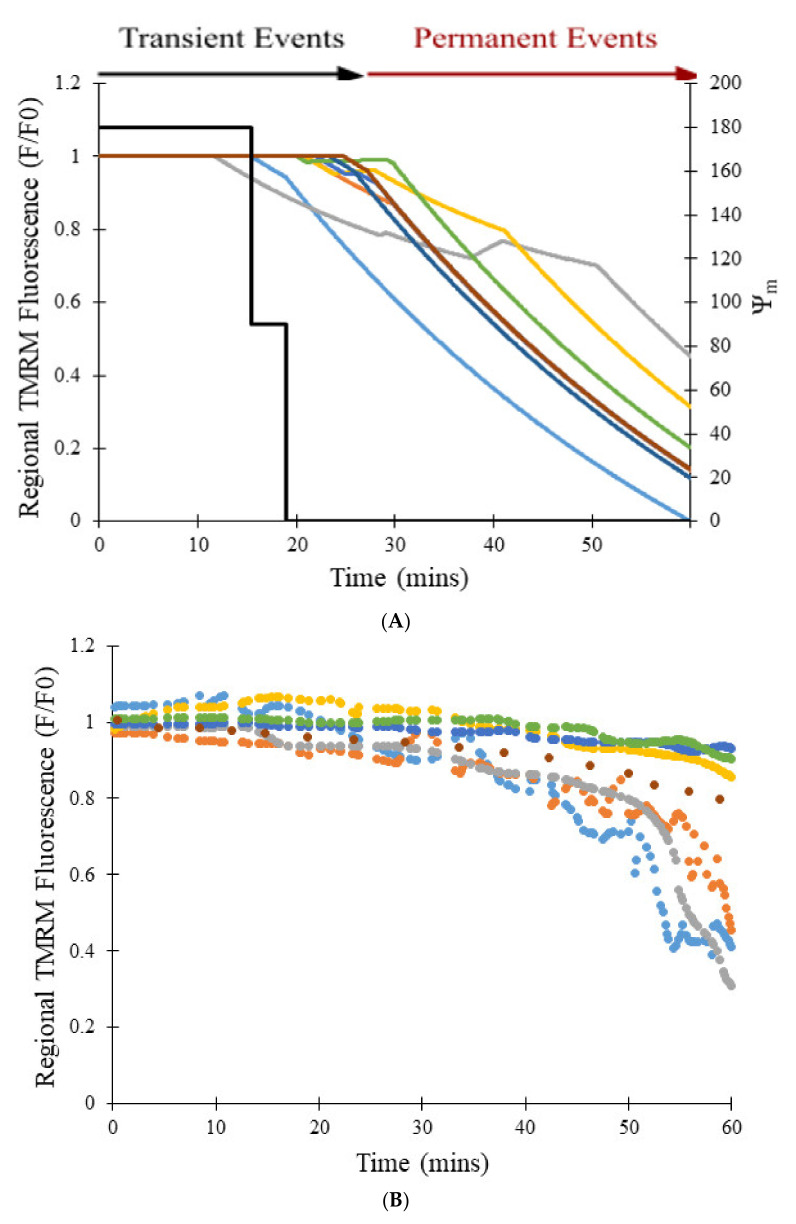

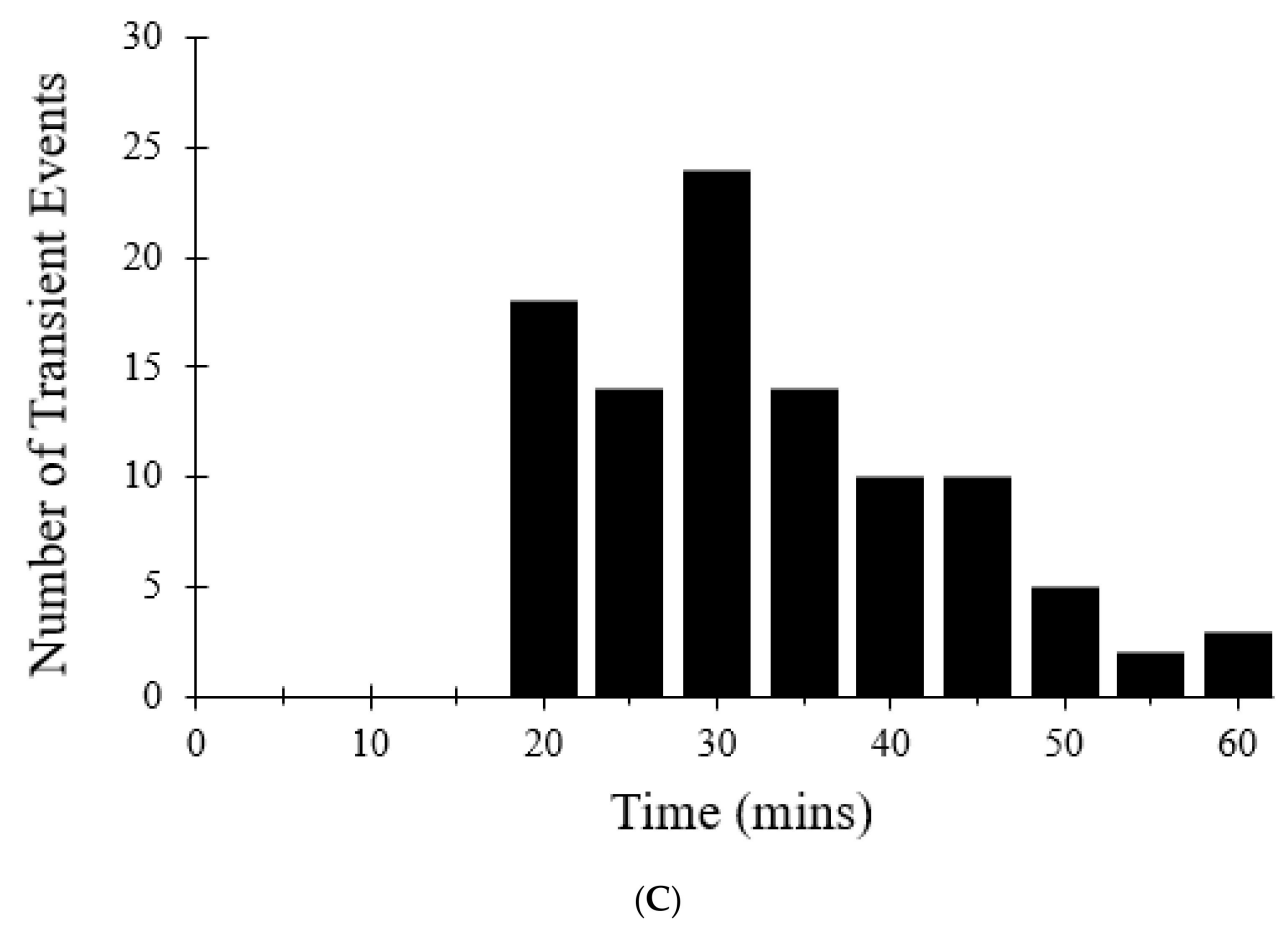
Transition from transient to permanent opening events under increased intensity of laser irradiation (photon stress). (**A**) Simulated regional TMRM fluorescence over time during transition from transient to permanent opening events. ROS levels were increased to replicate the effects of increased photooxidation on ROS production. Data was collected from 6 regions of interest inside the cell. Mitochondrial membrane potential during the transition from transient to permanent opening events with increased photon stress. (**B**) Regional experimental TMRM fluorescence over time during transition from transient to permanent opening events [7]. (**C**) Number of transient opening events per cell per minute with increased photon stress. A simulation of 100 pores was used to collect data.

Figure 9A shows the transition from transient to permanent opening events over time as photon stress increases in different cells, which models the experimental data represented in Figure 9B [7]. The transition rate to the permanent open state is proportional to the number of pores in the transient open state, as expected by Equation (8), and this dependence becomes steeper as [ROS] increases with time (in control and CsA simulations). In the model, photon stress increased ROS levels by the differential Equation (13), the rate at which mPTP transitions from the transient open state to the permanent open. Figure 9C shows the impact of increased photon stress on the TMRM fluorescence over 60 min. The number of simulated transient events decreases after ~30 min since there are fewer pores available to make the transition due to the accumulation in the permanent open state.

**Figure 10:**
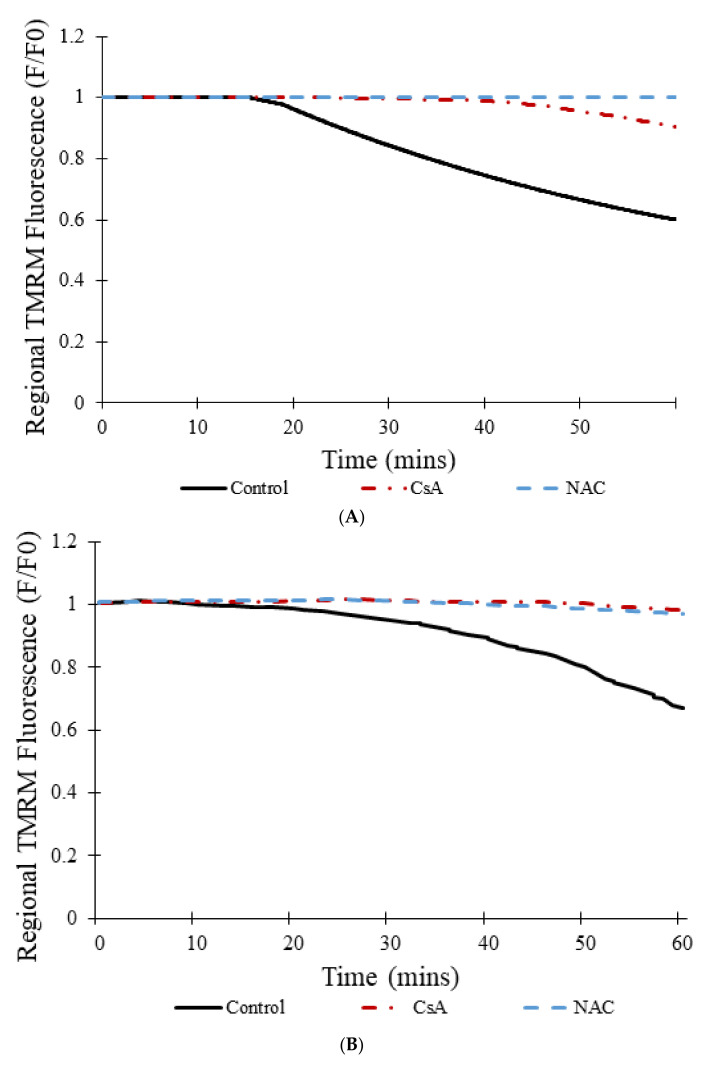

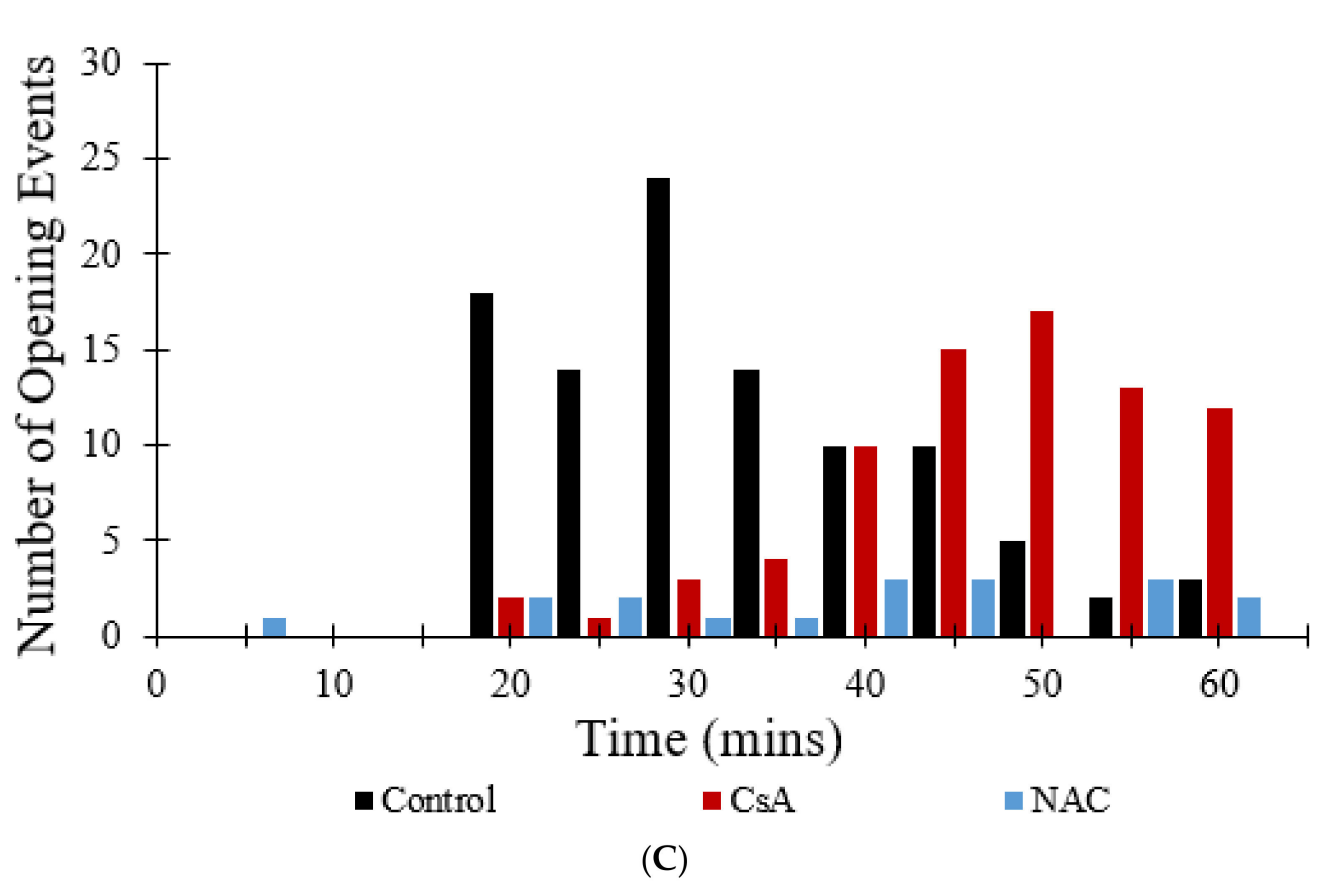
Transition from transient to permanent opening events under increased photon stress for CsA, NAC, and control groups. (**A**) Simulated regional TMRM fluorescence over time during transition from transient to permanent opening events in the CsA, NAC, and control groups. (**B**) Average experimental TMRM fluorescence over time during transition from transient to permanent opening events in the CsA, NAC, and control groups [7]. (**C**) Number of simulated transient and permanent opening events per cell per minute with increased photon stress in the CsA, NAC, and control groups. A total of 100 pores were simulated to produce the data in all cases.

Figure 10A shows the transition from transient to permanent opening events over time for models as photon stress increases in cells from different groups: control, CsA, and NAC, similar to the experimental data shown in Figure 10B [7]. Over time, the average TMRM fluorescence decreased in cells from all groups. However, a clear distinction exists between the control group and the two experimental groups (CsA and NAC). Whereas the average TMRM fluorescence decreased by almost 40% over the course of 60 min in the control group, the decrease was significantly less in the groups with CsA or with NAC. Figure 10C shows that the addition of CsA or NAC significantly decreases the number of simulated transient and permanent opening events with increased photon stress. In the control, as more mPTP pores enter the permanent open state, the number of openings decreases since there are fewer pores available.

**Figure 11:**
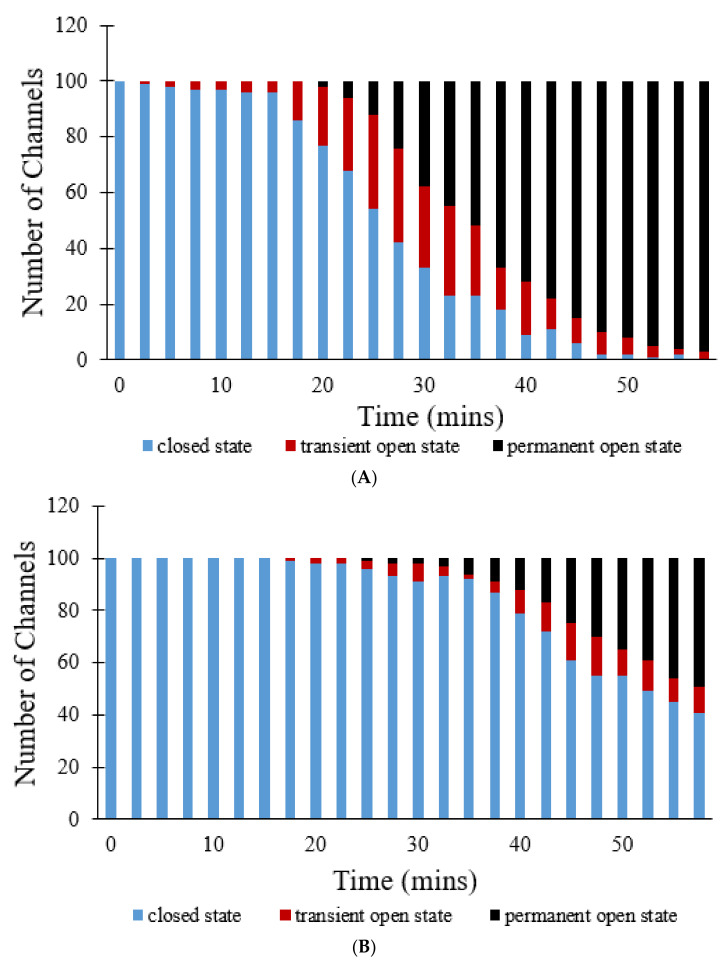

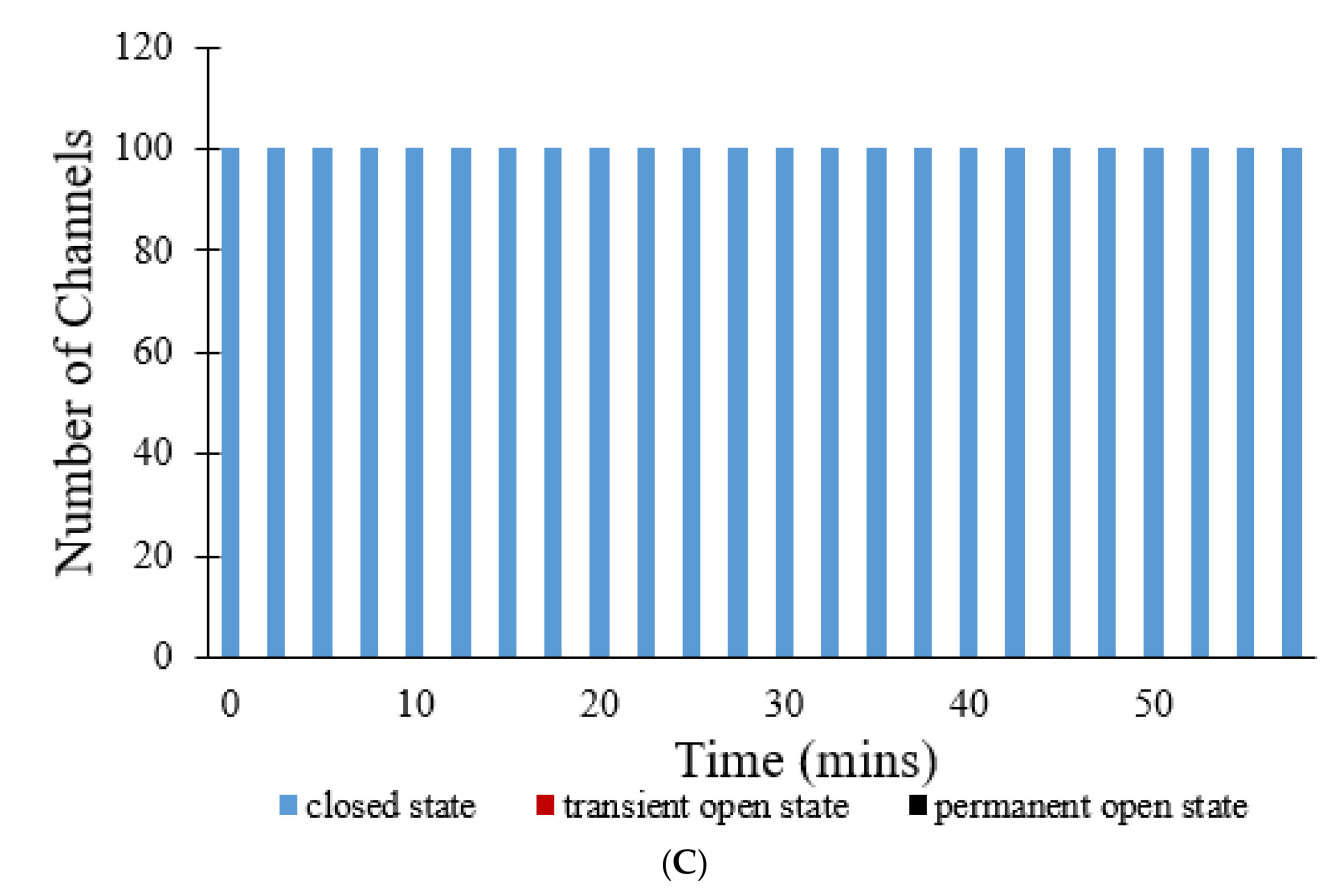
Transition between closed state, open state, and permanent open state for CsA, NAC, and control groups for simulations involving 100 mPTPs. (**A**) Number of pores in each of three different states (closed, transient open, and permanent open) over time. (**B**) Number of pores in each of three different states (closed, transient open, and permanent open) over time with the addition of CsA. (**C**) Number of pores in each of three different states (closed, transient open, and permanent open) over time with the addition of NAC.

Figure 11 show simulations of 100 pores that all start off in the closed state. In Figure 11A, a steep decline in the number of pores in the closed state occurs at approximately the 18 min mark. These pores instead switch to the transient open state and the permanent open state. By the end of the 60 min simulation, no pores remain in the closed state, 3 pores are in the transient open state, and the remaining 97 pores have transitioned to the permanent open state. This occurs due to the fact that, with time, ROS levels increase, driving a majority of the pores to eventually enter the permanent open state due to the positive-sloped ROS dependence of the opening rate. The addition of CsA delays the transition from the closed state to the transient open state, which can be seen in Figure 11B. Here, the initial steep decline in the number of pores in the closed state is only initiated after about 38 min. Even after 60 min, 41 pores remain in the closed state, 10 pores are in the transient open state, and 49 pores have transitioned to the permanent open state. The addition of NAC resulted in even greater differences in the overall progression of the states of pores over time, as indicated in Figure 11C. In this figure, no transition can be seen at all, as all 100 pores continue to stay in the closed state over all 60 min, suggesting that NAC has significantly decreased ROS levels, and subsequently inhibited mPTP openings to an even greater extent than CsA. 

The final set of studies was designed to gain additional insight into possible mechanisms behind the dynamics of mPTP opening. The model presented above faithfully simulates the experimental data, however, all these studies involved one mPTP per mitochondrion. While the permanent open state for the pore can exist, it is also possible that there are multiple pores and that during sustained opening at least one of the pores remains open. To evaluate this alternative hypothesis, a model with multiple pores per mitochondrion was developed with each pore assuming only the closed and transient open state as follows:

(14) $$\mathrm{Closed}\;\mathrm{State}\underset{Q}{\overset{P}{⇌}}\;\mathrm{Transient}\;\mathrm{Open}\;\mathrm{State}\;$$

where the transition probabilities P and Z are defined similarly as above with the factor 1/n added to the opening rate to keep the number of opening events relatively consistent as the number of channels changes

(15) $$P=\frac{1}{n}\frac{8.25\times 10^{-7}{\left[{\mathrm{Ca}}^{2+}\right]}_{e}^{3}}{{\left[96\right]}^{3}+{\left[{\mathrm{Ca}}^{2+}\right]}_{e}^{3}}\frac{{\left[\mathrm{ROS}\right]}^{4}}{0.1^{4}+{\left[\mathrm{ROS}\right]}^{4}}\mathrm{dt}$$

(16) Q=0.1dt

**Figure 12:**
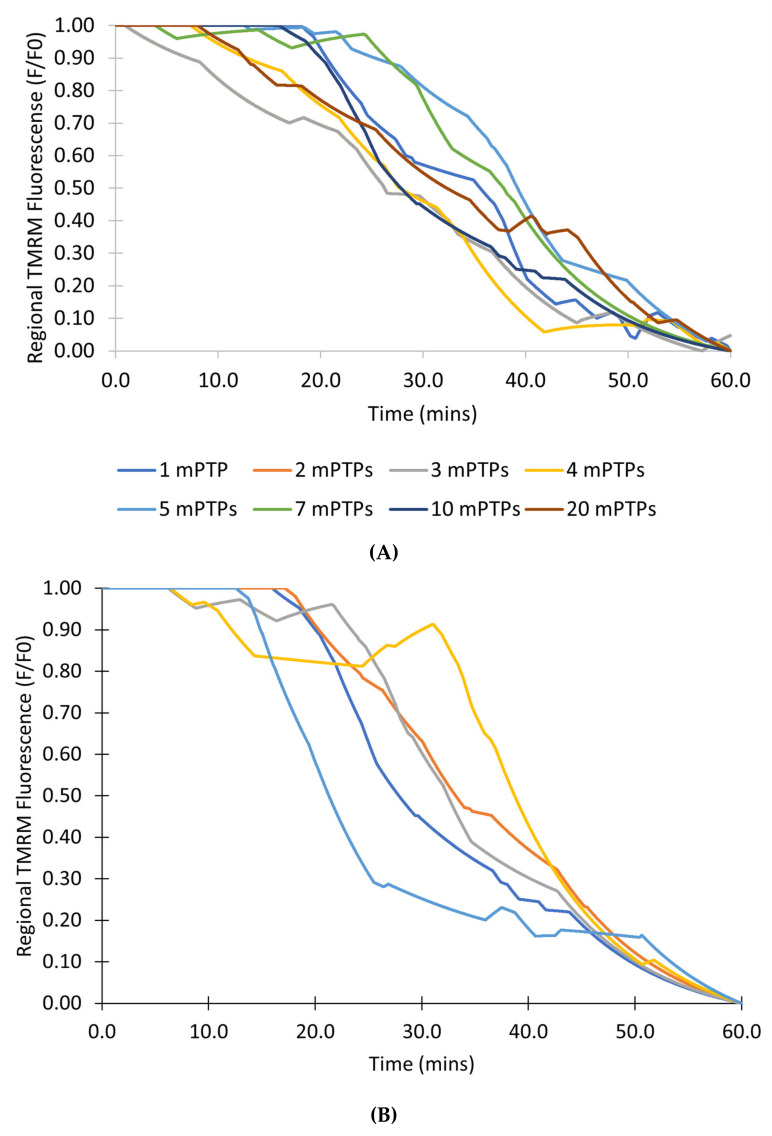
Simulation in the mPTP model with only transient states. (**A**) Simulations showing the average TMRM fluorescence from 100 mitochondria with 1, 2, 3, 4, 5, 7, 10, and 20 pores each. (**B**) Five simulations showing the average TMRM fluorescence from 5 mitochondria with 10 pores each.

The modeling studies next explore the transition from transient to permanent events. The first set of simulations with this model explored how the number of channels affected simulation results. Figure 12A shows the simulations of 100 mitochondria with 1, 2, 3, 4, 5, 7, 10, and 20 pores. When at least one pore is open, the mitochondrion depolarizes. Therefore, when enough pores are transiently open so they cannot stochastically close at the same time, the permanent open state occurs. The number of pores does not seem to affect the transition to permanent open state. Additional simulations were performed with 10 pores in 100 mitochondria to address the variability of the transition to a permanent open state for 5 simulation runs with different random seeds (Figure 12B). There is variability in the transition similar to experiment and the previous model. The only observable differences are that there seems to be more positive inflection (caused by all the mPTPs in a mitochondrion being closed) than in experiment and the previous model. 

**Figure 13:**
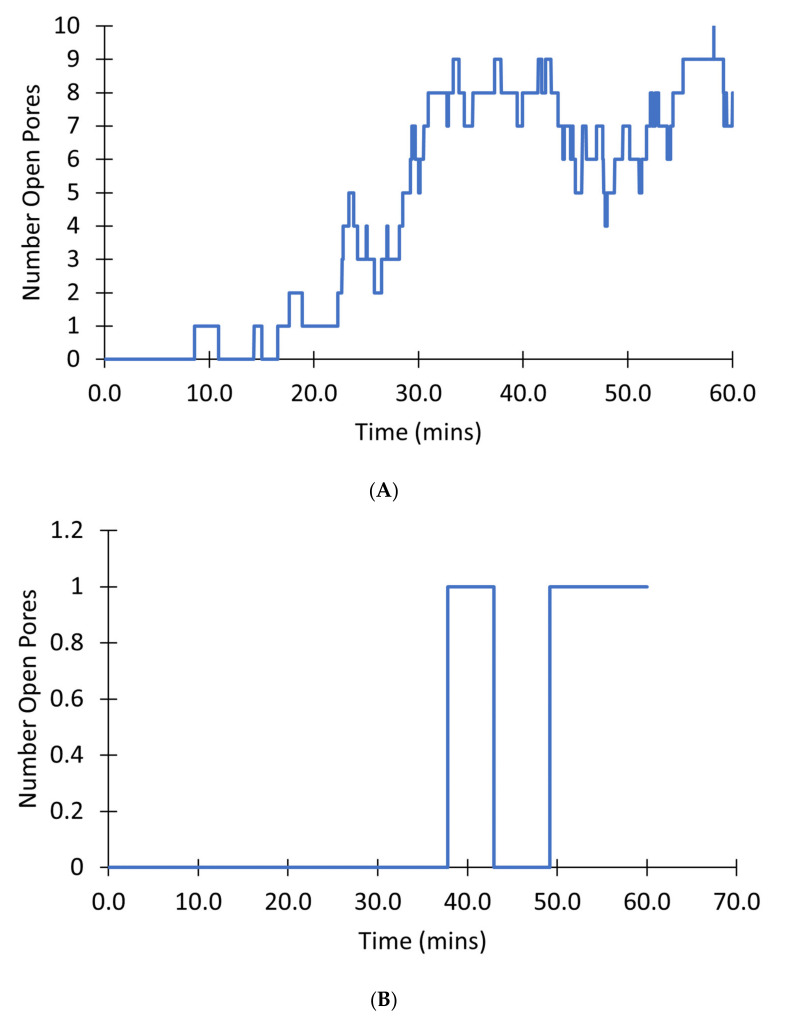
Simulation of a mitochondrion mPTP with only transient states. (**A**) Number of pores in the transient open state in a mitochondrion with 10 pores. (**B**) Number of open pores in the transient open state in a mitochondrion with 1 pore.

Figure 13 shows the number of pores in the transient open state for a single mitochondrion. In a mitochondrion with 10 pores, as the simulation progresses, and [ROS] increases there is almost always at least one channel open after 20 min as demonstrated in Figure 13A. By contrast, in a mitochondrion with only 1 pore as shown in Figure 13B, there can be times when there is no pore in the open state and the mitochondrion membrane potential can recover until around 50 min. Overall, such simulations indicate that once the number of pores is five or higher, the chance of all pores being closed is unlikely, in effect leading to a transition to a permanently depolarized state.

## 4. Discussion

This study examines the behavior of the mPTP through the development of a computational model replicating the results of an earlier experimental study by Boyman et al. [7]. The computational model used Markov Chain Monte Carlo simulation to simulate mPTP dynamics and ordinary differential equations to describe the ROS and TMRM dynamics. A Hill equation describes the dependence of the mPTP opening rates on ROS and Ca^2+^ with the parameters fit to match the experimental data. The main purpose was to clarify the interpretation of the experimental data and verify the accuracy of the conclusions drawn in the experimental study. The first model based on observed phenomena was able to simulate the experiments. 

The model assumes that ROS and Ca^2+^ are independent factors. Some recent studies have suggested that ROS is responsible for mPTP formation and Ca^2+^ is primarily responsible for opening [19]. Others have suggested that Ca^2+^-dependent opening of mPTP is modulated by ROS [20]. An alternate formulation of the opening transition is possible. For example, if the level of ROS modulates the Ca^2+^-dependent opening of mPTP the opening rate can be written as

(17) $$P=\frac{V_{\max}{\left[{\mathrm{Ca}}^{2+}\right]}_{e}^{m}}{{\left(K_{\mathrm{Ca}}\frac{K_{\mathrm{ROS}}{}^{n}}{K_{\mathrm{ROS}}{}^{n}+{\left[\mathrm{ROS}\right]}^{n}}\right)}^{m}+{\left[{\mathrm{Ca}}^{2+}\right]}_{e}^{m}}\mathrm{dt}$$

where V_max_ is the maximal rate, K_ROS_ is the EC_50_ for ROS, K_Ca_ is the EC¬_50_ for calcium, and n and m are Hill coefficients for the ROS and Ca^2+^ dependence, respectively. However, in the current study, which focuses on ROS dependent dynamics, the Ca^2+^ levels are not varied. Once sufficient additional quantitative data are gathered on the interplay of mPTP opening with Ca^2+^ and ROS levels, such a model can be developed.

The mPTP is generally thought to be a large conductance channel (on the order of 1 nS) with multiple subconductance states [21]. However, it is also possible that the smaller and larger conductances represent separate physical processes, as suggested recently [22]. Previous studies have suggested that fA currents are sufficient to depolarize mitochondria [23]. The current model does not make any assumptions about pore conductance, but does assume that, once a mPTP opens, the mitochondrion depolarizes. Given the small volume of the mitochondrion, even a single pore opening of 100 pS conductance is sufficient to quickly depolarize the mitochondria, as concluded in our previous studies [11]. Furthermore these previous studies suggest that such mitochondrial depolarizations would produce TMRM fluorescence profiles similar to those observed experimentally [11].

With regards to the idea that transient and permanent changes in fluorescence might represent separate processes, an alternative model can be developed involving different channels described as:

(18) $$\mathrm{Closed}\;\mathrm{State}\underset{Q}{\overset{P}{⇌}}\;\mathrm{Transient}\;\mathrm{Open}\;\mathrm{State}\;$$

(19) $$\mathrm{Closed}\;\mathrm{State}\overset{Z}{\to }\;\mathrm{Permanent}\;\mathrm{Open}\;\mathrm{State}$$

The formulations of P, Q, and Z would be similar to those in the current model with some adjustments to match the data. Such a model most likely could reproduce the experimental data. However, at this time we prefer the current, simpler model that explains the progressive nature of the data in terms of a transition (transient to permanent openings) in a single pore entity with increasing [ROS].

The simulations suggest that the sampling rate did have an impact on the amplitudes of the transient events in the experimental study, as illustrated in Figure 5. When a large sampling interval of 10 s was used in the computational model (Figure 5A), simulation results were similar to those of the experimental study. On the other hand, when the sampling rate was decreased to less than 0.1 s (Figure 5B), results were clearly different. Whereas the data in Figure 5A has the appearance of a roughly normal distribution, that in Figure 5B is skewed resembling exponential decay as expected for stochastic ion channel gating [24]. The experimental data miss a majority of the events, particularly those with shorter openings and lower magnitude depolarizations, as indicated by reduction in TMRM fluorescence. Thus, a higher sampling rate would be needed to determine whether events with shorter amplitudes and time spans occur and (as predicted by the modeling) account for most of the total transient opening events. However, while a faster sampling rate is possible, it would, it would entail more frequent laser irradiation and change the dynamics of the system through increase ROS production. The net result would be more sustained depolarization events. The model also predicted that, as the number of mitochondria with mPTP in the permanent open state increases, the rate of transient openings should decrease due to fewer mitochondria with closed mPTP and intact membrane potential (Figure 9C and Figure 10A).

The computational model replicated the experimental study accurately in several other aspects. As shown in Figure 4, the computational model was fit to the experimental data on the average TMRM fluorescence values over time for transient opening events. Figure 6, Figure 7, and Figure 8 also show the similarities between the computationally derived data and the experimental data, which indicates that the simulation accounted for major variables in the experimental data. Best estimates of the dynamics of [ROS] were made in the computational model. If the [ROS] rise is steeper or less steep, adjustments would need to be made for the ROS sensitivity of the transition rate between the states of the mPTP. It is likely that the [ROS] increase is not as steady as assumed in the model due to the pulse applications of the laser. If experimental [ROS] were measured, a more sophisticated model of ROS dynamics would be plausible and the ROS sensitivity of mPTP could be modified accordingly. This improved model could include more of the complexities of ROS removal processes. The model did not explore the effect of [Ca^2+^] since although several studies have observed calcium dependence, there have not yet been thorough measurements of calcium dependence and robust quantitative data are not available. Most studies use high levels of laser stimulation, which has been shown by Boyman et al. (2019) [7] to obscure physiological behavior. Data collected using methods similar to Boyman et al. (2019) [7], albeit at different calcium levels, would help to model the calcium dependence of mPTP behavior. 

In order to get a better understanding of mPTP dynamics, a second model in which there were no permanent open states was presented in Figure 12 and Figure 13. As [ROS] rises the opening rate of the pore increases so that the probability of at least one pore being open is high. In this case the mitochondrion will remain depolarized. If there are only a few channels it is possible that all the channels will close resulting in repolarization and an increasing TMRM signal. For five or more channels this is unlikely. Under the assumption that the depolarization does become permanent without an increase in flickering before this transition suggests that there are at least 5 mPTP per mitochondria. There are probably less than 10 pores since a larger number of pores would lead to excessive flickering before transition to the permanent state. This estimate of 5–10 mPTPs per mitochondrion is consistent with estimates in the experimental literature [25]. Furthermore, experimental data tracking single mitochondria would be helpful to resolve this issue. The model assumes that all mPTP openings are independent, but there is probably some coupling due to the fact that as the mitochondrion depolarizes, the mechanism to replenish ROS removal is attenuated. This coupling would make the transition to the permanently depolarized state more switch-like. Future studies could explore this situation with a more detailed study of ROS dynamics.

The second model with multiple channels that undergo only transient openings is mathematically related to the first phenomenological model. If, instead of modeling separate stochastic ion channels, they are modeled with one Markov chain counting the number of open channels the model becomes

(20) $$0\;\mathrm{Open}\;\mathrm{States}\underset{Q}{\overset{\mathrm{nP}}{⇌}}1\;\mathrm{Open}\;\mathrm{State}\underset{2Q}{\overset{\left(n-1\right)P}{⇌}}2\;\mathrm{Open}\;\mathrm{States}\underset{3Q}{\overset{\left(n-2\right)P}{⇌}}3\;\mathrm{Open}\;\mathrm{States}..\underset{\mathrm{nQ}}{\overset{P}{⇌}}n\;\mathrm{Open}\;\mathrm{States}$$

where n is the number of pores. If the opening transition rates to the right of two open states are fast (perhaps due to some sort of coupling—maybe through ROS removal impairment), this becomes equivalent to the phenomenological model

(21) $$0\;\mathrm{open}\;\mathrm{states}\underset{Q}{\overset{\mathrm{nP}}{⇌}}1\;\mathrm{Open}\;\mathrm{State}\underset{2Q}{\overset{\left(n-1\right)P}{⇌}}\mathrm{more}\;\mathrm{than}\;1\;\mathrm{Open}\;\mathrm{State}$$

The model assumes that the action of CsA simply reduces the opening rate for each channel. The model does not explicitly use CsA binding sites. A more detailed model is possible, which includes the K_0.5_ and number of binding sites using mass action kinetics or steady-state approximation. Such a model can be constrained using data from the experimental literature [26]. However, in this research study, the current simplified approximation is sufficient.

There are ongoing studies to determine the molecular identity of mPTP, with various indications that ANT and ATP synthase contribute to the observed phenomena, with regulation by cyclophilin D, a protein “foldase” regulated by CsA [27]. The modeling in this study was agnostic to competing theories about molecular composition of mPTP, but rather modeled the overall behavior of mPTP in terms of the stochastic behavior of ion channels [28]. An expectation would be that with transient depolarizations caused by channel openings, ATP production would decrease until repolarization occurs. 

## 5. Conclusions

Combining computational models with experimental results greatly helps to clarify how different molecular mechanisms affect the data recorded experimentally. Here, the newly developed computational model for mPTP, based on minimal assumptions about channel behavior, accurately simulated experimental data. The model replicated many features of mPTP openings observed in cardiac myocytes triggered by “photon stress.” Importantly, it suggests that mPTP openings should follow an exponential open time distribution predicted by stochastic channel theory. Furthermore, the model suggests that for 5-10 mPTPs in each mitochondrion, transient openings can produce mitochondrial depolarization that mimics a single permanent opening. Finally, the model suggests how to vary conditions employed in experiments to garner further insights into mPTP dynamics.

## Figures and Tables

**Table 1:** Parameters in Computational Model.

Parameter	Definition	Value
dt	Amount of time between consecutive data points	0.1 s
[ROS]_0_	Initial concentration of ROS	0.01 µM
k_ROS_	Rate of laser induced ROS production	0.05 s^−1^
[BUF]_Total_	Amount of ROS buffering system	1 µM
K_BUF_	Equilibrium constant for ROS interaction with ROS buffering system	0.05 s^−1^
${\left[\mathrm{TMRM}\right]}_{\mathrm{Total}}$	Total amount of TMRM normalized concentration	1.0 µM
$k_{\mathrm{out}}$	Rate of exit of TMRM from mitochondria	0.1 s^−1^

## Data Availability

The model code is available in Appendix A.

## Appendix A

**Model Code**

implicit double precision (a-h,o-z)
dt = 0.01
time = 0.0
buftot = 1.0d0
aKbuf = 0.050
iseed = 86456
call srand(iseed)
do k = 1,50
iflag = 0
ros = 0.010d0
psi = 180.0d0
tmtot = 1.0d0
tmreon = 0.0002d0 * abs(psi)
tmreoff = 0.02d0
tmreinit = tmtot*tmreon/(tmreon+tmreoff)
tmre = tmreinit
istate = 0
time = 0.0
starttime = 0.0
endtime = 0.0
opentime = 0.0
do i = 1,3000
time = time + dt
bros = 1./(1.d0+(buftot*aKbuf)/((aKbuf+ros)**2))
ros = ros + dt*bros*(0.05)
f1 = 0.00250*ros
b1 = 0.1d0*dt
f2 = 1.d-3*dt*ros**4/(0.5**4+ros**4)
psi = 180*(1-0.5*dfloat(istate))
c         psi = 180.0d0
tmreon = 0.0002 * abs(psi)
tmre = tmre + dt*((tmtot-tmre)*tmreon - tmre*tmreoff)
r = rand()
if (istate.eq.0.and.r.le.f1) then
istate = 1
psi = 0.0d0
starttime = time
iflag = 1
goto 10
endif
if (istate.eq.1) then
if (r.le.f2) then
istate = 2
psi = 0.0d0
elseif (r.gt.f2.and.r.le.f2+b1) then
istate = 0
psi = 180.0d0
endtime = time
endif
endif
10  continue
write (6,*) time,istate,tmre,psi
c      write (6,*) ros,f2
enddo
20  continue
if (iflag.eq.1) then
opentime = starttime
c         write (6,*) opentime
endif
enddo
stop
      end
